# Network Pharmacology-Based Prediction of the Active Compounds, Potential Targets, and Signaling Pathways Involved in Danshiliuhao Granule for Treatment of Liver Fibrosis

**DOI:** 10.1155/2019/2630357

**Published:** 2019-07-03

**Authors:** Yueying Tao, Kunming Tian, Ji Chen, Danfeng Tan, Yan Liu, Yongai Xiong, Zehui Chen, Yingbiao Tian

**Affiliations:** ^1^The Affiliated Hospital of Zunyi Medical University, Zunyi 563000, China; ^2^School of Pharmacy, Zunyi Medical University, Zunyi 563003, China; ^3^Department of Preventive Medicine, School of Public Health, Zunyi Medical University, Zunyi 563003, China; ^4^Department of Pharmacy, Zunyi Second People's Hospital, Zunyi 563000, China; ^5^The Second Affiliated Hospital of Zunyi Medical University, Zunyi 563003, China

## Abstract

This study aims to predict the active ingredients, potential targets, signaling pathways and investigate the “ingredient-target-pathway” mechanisms involved in the pharmacological action of Danshiliuhao Granule (DSLHG) on liver fibrosis. Pharmacodynamics studies on rats with liver fibrosis showed that DSLHG generated an obvious anti-liver fibrosis action. On this basis, we explored the possible mechanisms underlying its antifibrosis effect using network pharmacology approach. Information about compounds of herbs in DSLHG was collected from TCMSP public database and literature. Furthermore, the oral bioavailability (OB) and drug-likeness (DL) were screened according to ADME features. Compounds with OB≥30% and DL≥0.18 were selected as active ingredients. Then, the potential targets of the active compounds were predicted by pharmacophore mapping approach and mapped with the target genes of the specific disease. The compound-target network and Protein-Protein Interaction (PPI) network were built by Cytoscape software. The core targets were selected by degree values. Furthermore, GO biological process analysis and KEGG pathway enrichment analysis were carried out to investigate the possible mechanisms involved in the anti-hepatic fibrosis effect of DSLHG. The predicted results showed that there were 108 main active components in the DSLHG formula. Moreover, there were 192 potential targets regulated by DSLHG, of which 86 were related to liver fibrosis, including AKT1, EGFR, and IGF1R. Mechanistically, the anti-liver fibrosis effect of DSLHG was exerted by interfering with 47 signaling pathways, such as PI3K-Akt, FoxO signaling pathway, and Ras signaling pathway. Network analysis showed that DSLHG could generate the antifibrosis action by affecting multiple targets and multiple pathways, which reflects the multicomponent, multitarget, and multichannel characteristics of traditional Chinese medicine and provides novel basis to clarify the mechanisms of anti-liver fibrosis of DSLHG.

## 1. Introduction

Liver fibrosis is a common pathological feature of chronic liver disease and a main contributor in the development of cirrhosis caused by chronic liver injury [1, 2]. It is characterized by excessive deposition of collagen-based extracellular matrix (ECM) in the liver and abnormal liver function. Various chemical/microbial factors could contribute to the occurrence of hepatic fibrosis, including alcohol, virus, drugs, toxicants, and autoimmune process [3–6]. Previous studies [7, 8] indicated that liver fibrosis, especially in early stage, could be reversed, while once the liver fibrosis develops into cirrhosis stage, it could not. Thereby, more and more scholars are working to find novel drugs that can effectively reverse or delay the development of hepatic fibrosis.

Since the 1970s, the traditional Chinese medicine(TCM) have been used for the treatment of liver fibrosis. Cumulating data have shown that the TCM compound possesses obvious advantages in treatment of liver fibrosis. However, due to the multicomponent, multitarget, and multichannel characteristics of TCM and their complex formulations, the detailed medicinal ingredients of Chinese medicines and their complex models of action are unclear, which impeded the modernization and clinical application of TCM [9–11]. In recent years, network pharmacology based on systems biology and polypharmacology is emerging. Network pharmacology explores the relevance of drug-disease from a holistic and systematic perspective, emphasizes the transition of research mode from “single target” to “multiple targets,” and systematically expounds the principles and rules of interaction between organism and drugs. As a powerful tool for exploring the potential mechanisms of various actions of TCM formulas and novel active ingredients via constructing a network of interactions between drugs and genes, targets and diseases, it has been widely used in TCM research and became a new approach for the development of TCM [10–12].

Danshiliuhao Granule (DSLHG, composed of* Lysimachiae Herba*(LH),* Polygoni Cuspidati Rhizoma Et Radix*(PC),* Radix et Rhizoma Rhei *(RR),* Gardeniae Fructus*(GF),* Aurantii Fructus*(AF),* Aucklandiae Radix*(AR),* Corydalis Rhizoma*(CR), and Sodium Sulfate(SS)) is a Chinese herb preparation based on TCM prescription with wonderful anti-liver fibrosis effect and preservation of liver function. Our work provided theoretical basis for the registration of pharmaceutical preparations and laid the foundation for the subsequent acquisition of new medicine certificates. Among these herbal medicines, RR is a commonly used TCM. Many studies have proven that emodin has good clinical value in the treatment of liver fibrosis [13–15]. The rhein possesses anti-inflammatory and free radical scavenging effects and antifibrosis effect, protects liver cells, and reduces liver damage [16]. Additionally, PC exerts antibacterial, antiviral, and liver protection effect that can promote liver cell regeneration and recovery. Thereby, the beneficial effects of PC make it have therapeutic effect on acute hepatic ischemic injury, as evidenced by abrogated acute liver injury caused by carbon tetrachloride (CCl_4_) [17, 18]. In our study, we found that DSLHG could alleviate liver fibrosis induced by CCl_4_ in rats and raised one new hopeful drug for the treatment of hepatic fibrosis. However, due to its complex components and molecular mechanisms against liver fibrosis, which have not been fully elucidated, it is of great significance to explore the anti-liver fibrosis mechanisms of DSLHG through network pharmacology.

**Figure 1:**
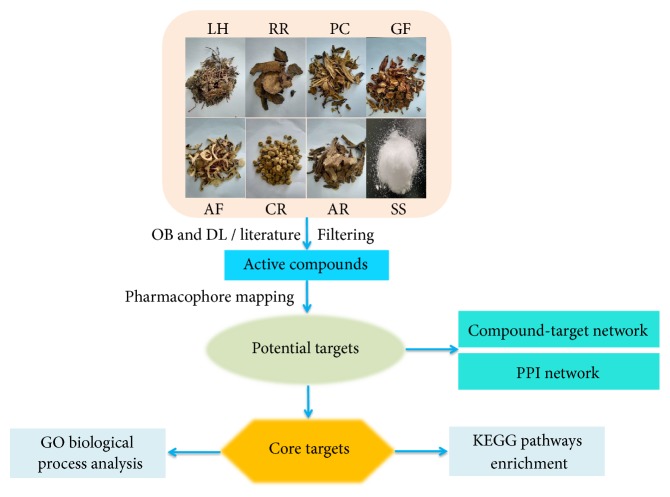
The schematic illustration of network pharmacology analysis.

In this work, we investigated the pharmacological mechanisms by which DSLHG regulates liver fibrosis. For this purpose, we established and analyzed active compound-target(C-T)network. First of all, we selected the active ingredients according to pharmacokinetic parameters (oral bioavailability and drug-likeness) from public database and searched the potential targets of these compounds by PharmMapper server. Furthermore, we built a network of interactions among active components regarding their related-targets and target genes of liver fibrosis. Moreover, we conducted GO function analysis and relevant pathway enrichment analysis for the potential core targets of DSLHG acting on targeted disease. The schematic illustration of this study is shown in Figure 1.

## 2. Materials and Methods

### 2.1. Reagents and Chemicals

Silibinin capsules were provided by Tianjin TASLY SANTS Pharmaceutical CO., Ltd. (Tianjin, China). CCl_4_ was obtained from Shanghai Aladdin Bio-Chem Technology Co., Ltd. (Shanghai, China). Olive oil was purchased from Shanghai Yien Chemical Technology Co., Ltd. (Shanghai, China). CCl_4_ was diluted to 40% (v/v) with olive oil before being used. The detection kits of alanine aminotransferase(ALT), aspartate aminotransferase (AST), alkaline phosphatase (ALP), and albumin (ALB) were purchased from the Nanjing Jiancheng Bioengineering Institute (Nanjing, China). Enzyme-linked immunosorbent assay(ELISA) kits for the measurement of laminin(LN), hyaluronic acid(HA), procollagen-III-peptide (PIIINP), and collagen type IV (ColIV) were offered by Shanghai Jiang Lai Biological Technology Co., Ltd. (Shanghai, China).

### 2.2. Animals and Experimental Design

Sixty healthy male Sprague Dawley (SD) rats weighting 180-220g were purchased from the Changsha Tianqin Biotechnology Co., Ltd. (Changsha, China). All the animals were housed under the same controlled conditions with a 12 h light-dark cycle at 23±2°C temperature in the Laboratory Animal Center of Zunyi Medical University (Guizhou, China). Then, the rats were randomly divided into 6 groups with 10 rats in each group: named (I): normal control group,(II): model group, (III): positive control group (Silibinin 36mg/kg·d^−1^), (IV): DSLHG high-dose group (27g/kg·d^−1^), (V): DSLHG medium-dose group (13.5g/kg·d^−1^), (VI): DSLHG low-dose group (6.75g/kg·d^−1^). All groups were given normal water and standard rat feed. From the first day of the experiment, the abdominal cavities of other rats were injected 40% CCl_4_ with olive oil (0.2mL per 100gof bodyweight) twice a week (Tuesday and Friday) except for the normal control group, and the normal rats were handled by intraperitoneal injection with olive oil solution(2mL/kg) for seven weeks. From the 2th week, the positive control group was intragastrically administered with a dose of 10mL/kg body weight of silibinin, and the DSLHG groups were gavaged with 10mL/kg body weight of the corresponding drug concentration. In addition, the normal control group and the model group were given a gavage of distilled water of the same volume with the drug. Administration of all animals was continued for six weeks.

At the end of the eighth week, all rats were anesthetized by ether after the last gavage. Then, blood samples were collected from the rat eyelid by capillary. The whole blood was centrifuged at 3500rpm for 10min, and the upper serum was taken in a cryogenic vial and then immediately stored in a refrigerator at -20°C to test related biochemical indicators using corresponding kits. The liver tissues of the rats were removed rapidly and rinsed in pre-cold physiological saline. The right lobes of the liver (approximately 1×1×1cm^3^) were quickly fixed in 10% neutral formaldehyde solution for histopathological examination.

This protocol was approved by the Animal Care and Use Committee of the Zunyi Medical University (2019A-002) and all experimental procedures were in compliance with the National Institutes of Health Guide for Care and Use of Laboratory Animals.

### 2.3. Histopathological Examination

The fixed liver tissues with 10% neutral buffered formaldehyde solution were dehydrated by automatic dehydrator, embedded in paraffin, and then sliced to a thickness of 4-6*μ*m sections. Hematoxylin-eosin (H&E) staining and Masson's trichrome staining were performed to observe the pathological and fibrosis changes, respectively. The degree of inflammation was graded based on the METAVIR scoring system: A0: no activity; A1: mild activity; A2: moderate activity; A3: severe activity. The degree of liver fibrosis was graded on a 5-point scale as either F0, F1, F2, F3, or F4, i.e., no fibrosis, portal fibrosis without septa, portal fibrosis with few septa, numerous septa without cirrhosis, and cirrhosis.

### 2.4. Chemical Ingredients Database Construction

Information of chemical compounds of each herbal medicine in DSLHG was obtained from free public database and literature. Traditional Chinese Medicine Systems Pharmacology Database and Analysis Platform [19](TCMSP, http://lsp.nwu.edu.cn/tcmsp.php), a unique system pharmacology platform designed for herbal medicines, provides a chemical screening process based on ADME features. According to the pharmacokinetic characteristics given by the TCMSP data platform, the chemical components that cannot satisfy both oral bioavailability (OB)≥30% and drug-likeness (DL) ≥0.18 are deleted [20]. The 2D structure of the candidate compound was searched by the PubChem database and drew with ChemBioDraw 14.0 software. Finally, the 2D structure was converted to 3D structure and saved as a MOL2 file.

### 2.5. Target Genes Related to the Identified Compounds

The target prediction for the main active compounds was performed using the PharmMapper server[21] (http://www.lilab-ecust.cn/pharmmapper/) with the “*Homo sapiens*” species setting, which is a free public web-server that uses active small molecules as a probe to search potential drug targets. The 10 targets with the highest Fit Score were selected, and gene information including gene ID, name, and organism was identified using UniProt database(https://www.uniprot.org/).

### 2.6. Potential Target Genes for Liver Fibrosis

The target genes associated with hepatic fibrosis were collected from the following resources: (1)GeneCards[22](https://www.genecards.org/) is a human gene database that automatically integrates data from approximately 150 web sources. We searched GeneCards database with the keywords “hepatic fibrosis” or “liver fibrosis” and found 1,222 genes related to hepatic fibrosis from the database. (2)The Online Mendelian Inheritance in Man(OMIM) database [23] is a continuously updated database of human genes and genetic disorders. We retrieved 50 hepatic fibrosis-related genes from the OMIM database. (3) Literature, we gathered liver fibrosis-related literature that contains 81 target genes. We finally collected a total of 1,280 genes associated with liver fibrosis through the above sources.

### 2.7. Construction of Network

In order to understand the mechanisms of DSLHG for the treatment of liver fibrosis, network analysis was performed. The corresponding network was established and visualized by Cytoscape 3.7 software. Nodes in the network represent active components and target genes. The edges indicate that the ingredients interact with the targets. The Protein-Protein Interaction (PPI) network of DSLHG on liver fibrosis was constructed using the STRING database (https://string-db.org/) combined with the Network Analyzer plugin of Cytoscape to filter out hub genes.

### 2.8. Pathway and Functional Enrichment Analysis

We carried out Gene Ontology (GO) function analysis and signaling pathway enrichment analysis for predicting targets of DSLHG on liver fibrosis by using DAVID bioinformatics resources 6.8 (https://david.ncifcrf.gov/).

### 2.9. Statistical Analysis

All experimental data were taken on the mean ± standard deviation (SD). Statistical analyses were performed with GraphPad Prism 6.0 statistical software. Rank data were analyzed with the rank sum test. A one-way analysis of variance (ANOVA) test was used to compare the mean of different groups. The data were compared by Student's* t *test between two groups, and* P*<0.05 was considered as statistically significant.

## 3. Results

### 3.1. Animal Experiments

**Figure 2:**
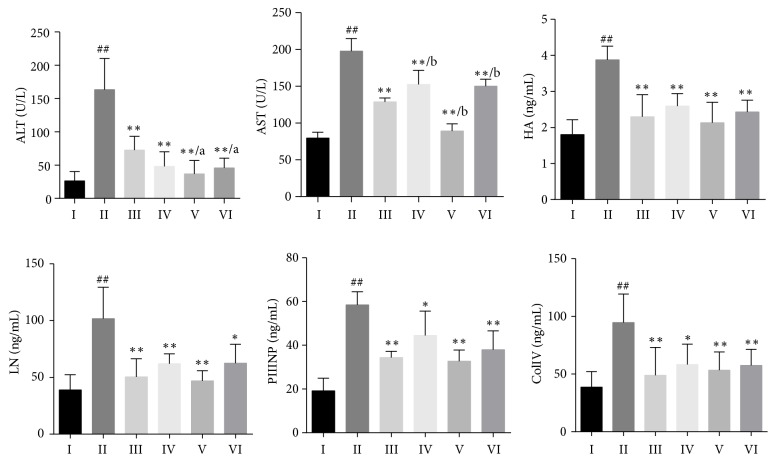
Effect of DSLHG on serum biochemical levels in CCl_4_-induced liver fibrosis rats (mean ± SD). ^##^*P*<0.01, compared with the normal control group; *∗P*<0.05, *∗∗P*<0.01, compared with the model group; ^a^*P*<0.05, ^b^*P*<0.01, compared with the positive control group. I: the normal control group; II: the model group; III: the positive control group; IV: DSLHG high-dose group; V: DSLHG medium-dose group; VI: DSLHG low-dose group.

The results of serum biochemical indicators in rats are shown in Figure 2. Compared with the model group, ALT, AST, HA, LN, PIIINP, and Col IV levels in rat serum were significantly reduced in DSLHG groups, as well as in the positive control group (*P*<0.05 or* P*<0.01).

**Figure 3:**
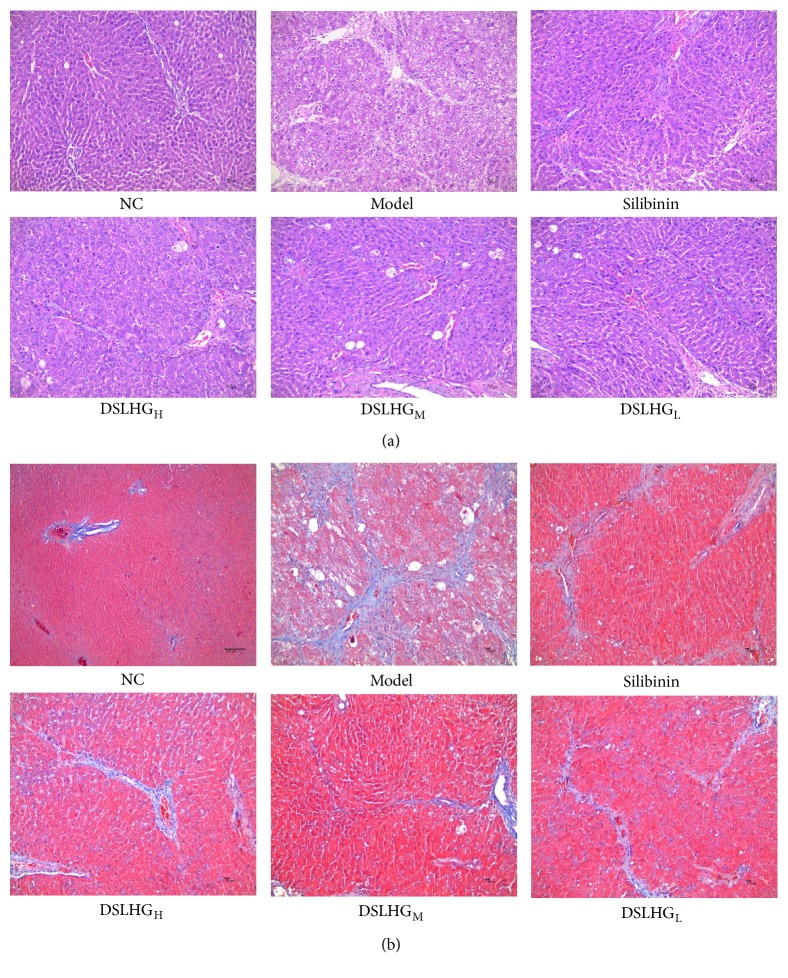
Histological results of liver tissues for each group. (a) HEstaining (×200); (b) Masson's trichrome staining(×200). NC: the normal control group; Model: the model group; Silibinin: the positive control group; DSLHG_H_: DSLHG high-dose group; DSLHG_M_: DSLHG medium-dose group; DSLHG_L_: DSLHG low-dose group.

HE and Masson staining of liver sections were shown in Supplementary Figure 1. As shown in Figure 3, in normal control group, the hepatic lobule structure was clear, and hepatic cords were arranged radially around the central veins, and only a small amount of blue collagen fiber deposition was observed, while the liver tissue of model group indicated thickened capsule, destroyed or disappearing partial normal hepatic lobular structure, and structurally appearing “pseudolobule.” Compared with model group, the histopathological examination showed that collagen deposition, liver inflammation activity, and liver fibrosis significantly reduced after treatment with DSLHG and silibinin. Additionally, no obvious pseudolobules were observed.

As shown in Table 1, rank sum test showed statistical difference in the pathological and fibrosis changes between all treatment groups and the model group(*P*<0.05). Unfortunately, none of the DSLHG groups were significantly different from the silibinin group in either of these parameters (*P*>0.05). These results showed that DSLHG had an obvious anti-liver fibrosis effect.

### 3.2. Active Ingredients Filtering

Total of 513 chemical constituents of seven individual herbs(Sodium Sulfate was not found in the database) in DSLHG were retrieved from TCMSP, including 61 ingredients in LH, 92 in RR, 62 in PC, 98 in GF, 17 in AF, 106 in AR, and 77 in CR. All compounds were subjected to ADME screening, and a total of 111 active compounds had OB≥30% and DL≥0.18. There are 10 compounds in LH, 16 compounds in RR, 10 compounds in PC, 15 compounds in GF, 5 compounds in AF, 6 compounds in AR, and 49 compounds in CR, respectively. Due to duplication, fifteen of these compounds were deleted, resulting in 96 effective active ingredients. In addition, among the compounds below the OB and DL screening criteria, 12(emodin, chrysophanol, physcion[24–26], resveratrol[27], polydatin[28], geniposide, genipin, geniposidic acid, genipin 1-gentiobioside[29–32], dehydrocostus lactone[33], neohesperidin[34], and tetrahydropalmatine[35]) were considered to be biologically active compounds, and their protective effect on liver has been reported. Therefore, the selected 108 compounds (Supplementary Table 1) from DSLHG formula were subjected to further analysis.

### 3.3. Potential Targets in DSLHG

Top 10 targets with the highest Fit Score were selected. Total of 192 potential targets were obtained from 108 active ingredients after removing the redundancy (Supplementary Table 2), including MDM2, TNFBR1, MAPK14, and SRC, involved in inflammation, hepatic lipid metabolism, and the development of hepatic fibrosis. In order to make the targets more concentrated, we deleted 106 target genes which could not correspond to the disease. As shown in Table 2, 86 of 192 potential targets were associated with liver fibrosis.

### 3.4. Compounds-Targets(C-T) Network Construction and Analysis

**Figure 4:**
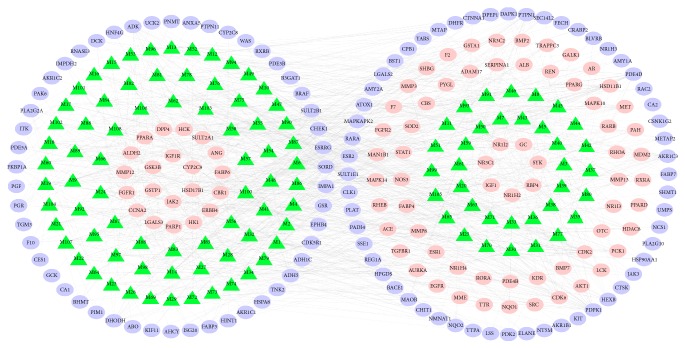
Potential active compound-target network of DSLHG acting on liver fibrosis. A compound node and a target node are linked if the protein is targeted by the related compounds. Green triangle nodes represent the active compounds of DSLHG; ellipse and circular nodes are the potential targets of DSLHG; the overlap of the potential targets of the active compounds in DSLHG formula and the liver fibrosis-related targets is indicated with pink circular nodes.

As shown in Figure 4, the 108 active compounds and related 192 target genes constructed the network schematic diagram. Totally, this C-T network is composed of 300 nodes (108 active compounds and 192 potential targets) and 1080 edges. In the picture, the edges indicate an association between the active ingredients and the targets. The degree values indicate the intensity of the interaction between the components and the targets. Green triangle nodes represent the active compounds of DSLHG. Circular nodes represent the potential targets of DSLHG, and the overlaps between the potential targets of the DSLHG and the liver fibrosis-related targets are indicated with pink circular nodes.

According to the network, one target gene may be complicatedly linked to multiple active ingredients of different herbs, which suggest that these compounds might synergistically contribute to the pharmacological effect of the DSLHG in the treatment of hepatic fibrosis. In addition, different active ingredients can also target the same gene, reflecting its multicomponent, multitarget, and antifibrosis effect, that was consistent with the multicomponent and multitarget characteristics of TCM.

### 3.5. PPI Network Construction and Analysis

**Figure 5:**
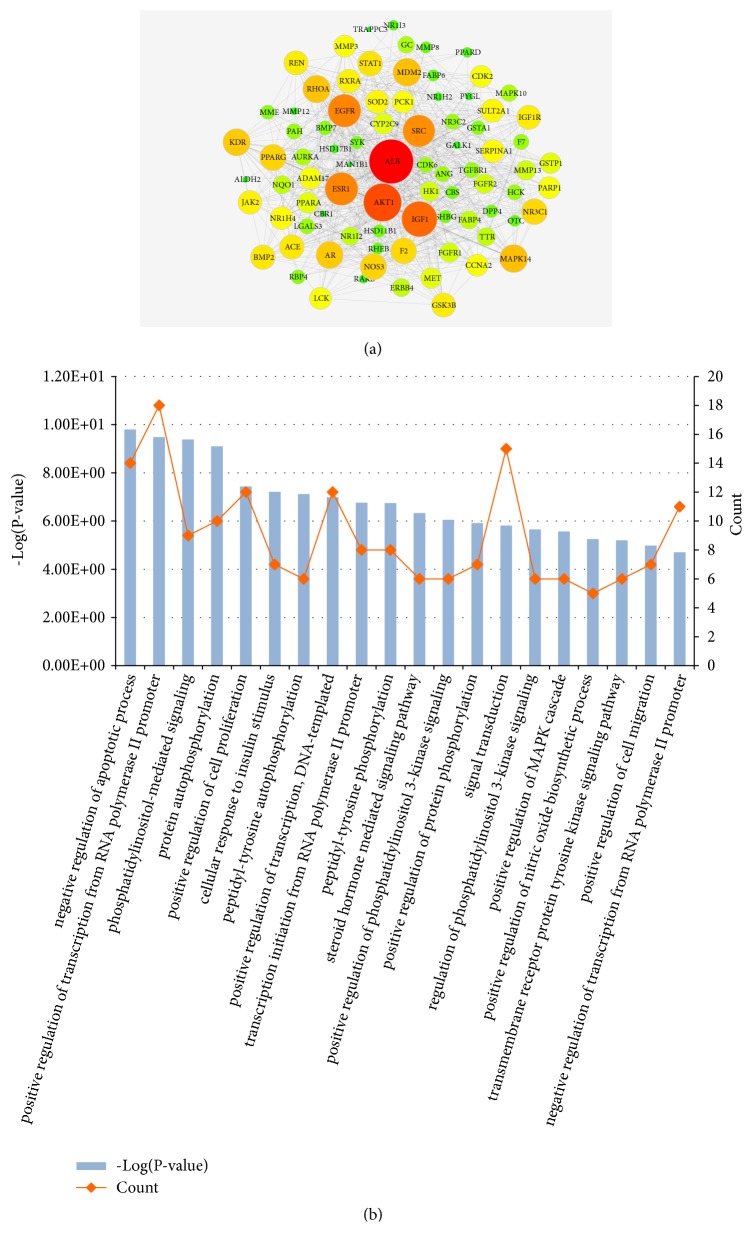
The protein interaction of overlap of the potential targets of the chemical components in DSLHG with the liver fibrosis-related target genes and annotation analysis of core genes. (a) The PPI network of 86 overlap genes. (b) GO function analysis for hub genes.* Notes.* The nodes were overlap protein genes; the size of nodes from small to large represent the degree value from low to high; and these nodes color changes from green to red to indicate that the degree values change from low to high.

86 target genes associated with active ingredients and liver fibrosis were imported into the STRING database for PPI network construction and analysis. There are 83 interacting targets in the network (HDAC8, RORA, and PDE4B are not involved in protein interaction), resulting in 594 edges representing the interaction between proteins (Figure 5(a)). Then, the PPI network was screened according to the degree values. In this work, the target genes with a degree value greater than 10 were selected as the core genes for further analysis and a total of 47 targets were screened out.

### 3.6. GO Functional Annotation Analysis

A total of 47 key potential genes that may be involved in liver fibrosis were uploaded to the DAVID panel for analyzing their biological process. The functional distribution of 47 targets was explored by GO functional analysis. The results of GO analysis of the predicted key targets of DSLHG acting on liver fibrosis are shown in Figure 5(b), which listed the 20 GO terms with low* P* values and more targets enrichment. The results showed that these targets have a strong association with physiological mechanisms, such as signal transduction, protein autophosphorylation, and positive regulation of cell proliferation. These results indicated that DSLHG may play a key role in antihepatic fibrosis by manipulating these biological processes which lead to the pathogenesis of liver fibrosis.

### 3.7. Kyoto Encyclopedia of Genes and Genomes (KEGG) Signaling Pathway Analysis

**Figure 6:**
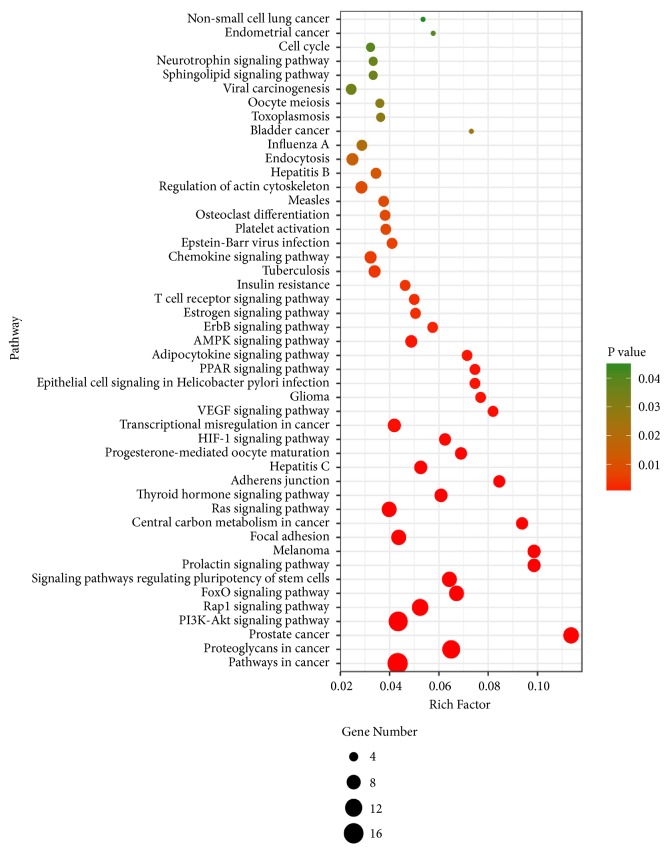
KEGG pathway enrichment analysis for core targets of DSLHG acting on liver fibrosis.

To determine the relevant signaling pathways involved in the anti-liver fibrosis effect of DSLHG, we conducted pathway enrichment analysis using KEGG pathways. A total of 47 targets obtained 60 KEGG signaling pathways, and 47 channels were significantly enriched (*P*<0.05). The senior bubble map visually showed these significantly enriched pathways (Figure 6). The color and size of the nodes in the bubble graph were decided by the number of associated genes and the* P* values. The colors from green to red reflected the* P* values from high to low, and the size of the nodes indicated how many target genes are associated. The results of KEGG pathways enrichment analysis indicated the multiple channels and mechanisms of action of DSLHG against liver fibrosis.

**Figure 7:**
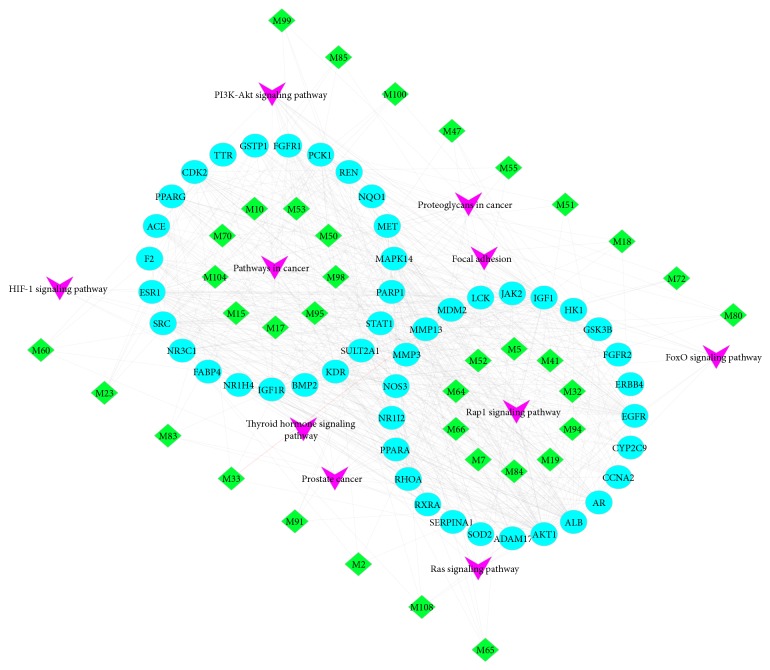
Main active compound(degree>3) core target-pathway network of DSLHG on treating liver fibrosis. Core target genes are shown with blue circular nodes; green diamond nodes represent the active compounds in DSLHG formula and purple V nodes are signaling pathways.

The top 10 pathways with lower* P *values and more genes enrichment are listed in Table 3, including PI3K-Akt signaling pathway, AMPK signaling pathway, Ras signaling pathway, T cell receptor signaling pathway, PPAR signaling pathway, VEGF signaling pathway, and FoxO signaling pathway being enriched. These signaling pathways involve inflammation, metabolism, and ECM deposition. Taken together, these signaling pathways seem to be closely related to the beneficial effects of DSLHG against liver fibrosis. The illustrated network that contains main chemicals-targets-signaling pathway of DSLHG was established to understand their interaction (Figure 7).

## 4. Discussion

Hepatic fibrosis is considered as the wound healing in response to the chronic liver damage caused by various factors. Excessive production and abnormal deposition of ECM are caused by the complicated interaction between various types of cells and cytokines in the liver, characterized by imbalance between the degradation and synthesis of ECM, resulting in a series of changes during the initiation and progression of liver fibrosis [3, 36, 37]. The occurrence of liver fibrosis is often accompanied by a certain degree of inflammatory response. Inflammation is one of the most typical features of viral, alcoholic, fatty, and autoimmune chronic liver disease. For a long time, many scholars have been working to explore the certain role of inflammation in the pathogenesis of liver fibrosis, such as the role of inflammatory mediators in the hepatic stellate cells (HSCs) activation and development of liver fibrosis [38]. HSCs are the main cells responsible for synthesis of ECM in normal and fibrotic liver and play central role in the development of liver fibrosis [39–41]. Once HSCs are activated through paracrine and autocrine routes, the imbalance between ECM synthesis and degradation was interfered, which results in aberrant accumulation of ECM in the liver [42, 43].

FoxO, a key downstream regulator in the PI3K-Akt signaling pathway belonging to subclass of the forkhead proteins family, regulates metabolic homeostasis in response to oxidative stress. Moreover, oxidative stress resistance, apoptosis, and glucose metabolism were also modulated by FoxO [44]. Importantly, FoxO transcription factors benefit liver fibrosis through inhibiting proliferation and transdifferentiation of HSCs [45].

As the downstream factor of the PI3K-Akt signaling pathway, FoxOs are controlled by Akt (protein kinase B, PKB). The PI3K/Akt signaling pathway is one of the important signaling pathways, which has increasingly raised concern under various diseases context. The PI3K-Akt signaling pathway can be activated by many types of cellular stimuli or toxic insults and regulates fundamental cellular functions, such as transcription, translation, proliferation, growth, and survival [46]. Some researchers [47, 48] have reported that PI3K and Akt are important in the process of the activation of hepatic stellate cells (HSCs), cell proliferation, and collagen synthesis. In contrast, PI3K and Akt are also involved in the regulation of HSCs apoptosis, which may be one of the important pathways delaying the development of liver fibrosis.

At present, most of the western medicines used for the treatment of liver fibrosis are effective only under a certain condition, with difficulty in achieving multitarget and multiechelon coverage. However, most diseases are associated with multiple targets; thus, it is difficult to achieve appropriate therapeutic results against a single target. The pharmacodynamic mechanisms of TCM against liver fibrosis have multiple levels and multiple targets and pay attention to the characteristics of overall regulation [10]. A new approach that analyzes TCM with network pharmacology may be a reliable way to overcome disease [49]. Cumulating data have shown that network pharmacology can reveal the interactions between multiple targets of compounds present in Chinese herbal medicines [50].

In this study, through the analysis of DSLHG compound-target-pathway network, we found that the main active components of DSLHG could act on multiple targets, indicating the multicomponent, multitarget, and overall regulation of formula. In addition, DSLHG generates the anti-liver fibrosis effect by acting on multiple pathways, multiple targets, and multiple biological processes. The predicted targets and signaling pathways in this study provide ideas for experiments to verify the key target proteins and mechanisms of antifibrosis of DSLHG in the future.

## 5. Conclusion

In summary, this study used a network pharmacology approach to construct a biological network to display the interactions between compounds and protein/gene targets at a molecular or systemic level. The results indicate that DSLHG can achieve antifibrosis by acting on multiple targets and multiple pathways. Through our network analysis and prediction, the mechanisms of DSLHG formula at the molecular level are clarified, which might provide new ideas for developing new drug treatment or novel therapeutical strategies for liver fibrosis. Next, we will verify the results of network analysis prediction through manipulating the targeted molecular via pharmacological or genetical methods.

## Figures and Tables

**Table 1:** Comparison of histological activity and fibrosis stages.

Groups	N	A				F				
		A0	A1	A2	A3	F0	F1	F2	F3	F4
Normal	6	2	4	0	0	3	3	0	0	0
Model	6	0	0	1	5^##^	0	0	0	2	4^##^
Silibinin	6	0	3	3	0*∗∗*	0	1	4	1	0*∗∗*
DSLHG_H_	6	0	2	3	1*∗∗*	0	0	4	2	0*∗∗*
DSLHG_M_	6	0	4	2	0*∗∗*	0	2	3	1	0*∗∗*
DSLHG_L_	6	0	2	4	0*∗∗*	0	2	4	0	0*∗∗*

A: activity grade, A0-A3; F: fibrosis stage, F0-F4. ^##^*P*<0.01, compared with the normal control group; *∗∗P*<0.01, compared with the model group.

**Table 2:** The overlapping targets for potential targets of the active compounds in DSLHG formula and the liver fibrosis-related targets.

No.	Protein name	Gene name	UniProt
P1	Angiotensin-converting enzyme	ACE	P12821
P2	Disintegrin and metalloproteinase domain-containing protein 17	ADAM17	P78536
P3	RAC-alpha serine/threonine-protein kinase	AKT1	P31749
P4	Serum albumin	ALB	P02768
P5	Aldehyde dehydrogenase, mitochondrial	ALDH2	P05091
P6	Angiogenin	ANG	P03950
P7	Androgen receptor	AR	P10275
P8	Aurora kinase A	AURKA	O14965
P9	Bone morphogenetic protein 2	BMP2	P12643
P10	Bone morphogenetic protein 7	BMP7	P18075
P11	Carbonyl reductase [NADPH] 1	CBR1	P16152
P12	Cystathionine beta-synthase	CBS	P35520
P13	Cyclin-A2	CCNA2	P20248
P14	Cyclin-dependent kinase 2	CDK2	P24941
P15	Cyclin-dependent kinase 6	CDK6	Q00534
P16	Cytochrome P450 2C9	CYP2C9	P11712
P17	Dipeptidyl peptidase 4	DPP4	P27487
P18	Epidermal growth factor receptor	EGFR	P00533
P19	Receptor tyrosine-protein kinase erbB-4	ERBB4	Q15303
P20	Estrogen receptor	ESR1	P03372
P21	Prothrombin	F2	P00734
P22	Coagulation factor VII	F7	P08709
P23	Fatty acid-binding protein, adipocyte	FABP4	P15090
P24	Gastrotropin	FABP6	P51161
P25	Fibroblast growth factor receptor 1	FGFR1	P11362
P26	Fibroblast growth factor receptor 2	FGFR2	P21802
P27	Galactokinase	GALK1	P51570
P28	Vitamin D-binding protein	GC	P02774
P29	Glycogen synthase kinase-3 beta	GSK3B	P49841
P30	Glutathione S-transferase A1	GSTA1	P08263
P31	Glutathione S-transferase P	GSTP1	P09211
P32	Tyrosine-protein kinase HCK	HCK	P08631
P33	Histone deacetylase 8	HDAC8	Q9BY41
P34	Hexokinase-1	HK1	P19367
P35	Corticosteroid 11-beta-dehydrogenase isozyme 1	HSD11B1	P28845
P36	Estradiol 17-beta-dehydrogenase 1	HSD17B1	P14061
P37	Insulin-like growth factor I	IGF1	P05019
P38	Insulin-like growth factor 1 receptor	IGF1R	P08069
P39	Tyrosine-protein kinase JAK2	JAK2	O60674
P40	Vascular endothelial growth factor receptor 2	KDR	P35968
P41	Tyrosine-protein kinase Lck	LCK	P06239
P42	Galectin-3	LGALS3	P17931
P43	Endoplasmic reticulum mannosyl-oligosaccharide 1,2-alpha-mannosidase	MAN1B1	Q9UKM7
P44	Mitogen-activated protein kinase 10	MAPK10	P53779
P45	Mitogen-activated protein kinase 14	MAPK14	Q16539
P46	E3 ubiquitin-protein ligase Mdm2	MDM2	Q00987
P47	Hepatocyte growth factor receptor	MET	P08581
P48	Neprilysin	MME	P08473
P49	Macrophage metalloelastase	MMP12	P39900
P50	Collagenase 3	MMP13	P45452
P51	Stromelysin-1	MMP3	P08254
P52	Neutrophil collagenase	MMP8	P22894
P53	Nitric oxide synthase, endothelial	NOS3	P29474
P54	NAD(P)H dehydrogenase [quinone] 1	NQO1	P15559
P55	Oxysterols receptor LXR-beta	NR1H2	P55055
P56	Bile acid receptor	NR1H4	Q96RI1
P57	Nuclear receptor subfamily 1 group I member 2	NR1I2	O75469
P58	Nuclear receptor subfamily 1 group I member 3	NR1I3	Q14994
P59	Glucocorticoid receptor	NR3C1	P04150
P60	Mineralocorticoid receptor	NR3C2	P08235
P61	Ornithine carbamoyltransferase, mitochondrial	OTC	P00480
P62	Phenylalanine-4-hydroxylase	PAH	P00439
P63	Poly [ADP-ribose] polymerase 1	PARP1	P09874
P64	Phosphoenolpyruvate carboxykinase, cytosolic [GTP]	PCK1	P35558
P65	cAMP-specific 3′,5′-cyclic phosphodiesterase 4B	PDE4B	Q07343
P66	Peroxisome proliferator-activated receptor alpha	PPARA	Q07869
P67	Peroxisome proliferator-activated receptor delta	PPARD	Q03181
P68	Peroxisome proliferator-activated receptor gamma	PPARG	P37231
P69	Glycogen phosphorylase, liver form	PYGL	P06737
P70	Retinoic acid receptor beta	RARB	P10826
P71	Retinol-binding protein 4	RBP4	P02753
P72	Renin	REN	P00797
P73	GTP-binding protein Rheb	RHEB	Q15382
P74	Transforming protein RhoA	RHOA	P61586
P75	Nuclear receptor ROR-alpha	RORA	P35398
P76	Retinoic acid receptor RXR-alpha	RXRA	P19793
P77	Alpha-1-antitrypsin	SERPINA1	P01009
P78	Sex hormone-binding globulin	SHBG	P04278
P79	Superoxide dismutase [Mn], mitochondrial	SOD2	P04179
P80	Proto-oncogene tyrosine-protein kinase Src	SRC	P12931
P81	Signal transducer and activator of transcription 1-alpha/beta	STAT1	P42224
P82	Bile salt sulfotransferase	SULT2A1	Q06520
P83	Tyrosine-protein kinase SYK	SYK	P43405
P84	TGF-beta receptor type-1	TGFBR1	P36897
P85	Trafficking protein particle complex subunit 3	TRAPPC3	O43617
P86	Transthyretin	TTR	P02766

**Table 3:** The important signaling pathways of core target genes.

Pathway ID	Term	Target genes	*P*-Value
hsa04151	PI3K-Akt signaling pathway	AKT1, EGFR, FGFR1, FGFR2, RXRA, MET, IGF1, CDK2, KDR, PCK1, IGF1R, GSK3B, MDM2, NOS3, JAK2	7.21E-0.9
hsa04068	FoxO signaling pathway	AKT1, EGFR, IGF1R, MAPK14, MDM2, IGF1, CDK2, PCK1, SOD2	1.14E-06
hsa04014	Ras signaling pathway	AKT1, EGFR, IGF1R, FGFR1, FGFR2, MET, RHOA, IGF1, KDR	5.39E-05
hsa04919	Thyroid hormone signaling pathway	AKT1, RXRA, GSK3B, ESR1, MDM2, STAT1, SRC	6.15E-05
hsa04015	Rap1 signaling pathway	AKT1, EGFR, FGFR2, IGF1R, FGFR1, MAPK14, MET, RHOA, IGF1, SRC, KDR	3.55E-07
hsa04510	Focal adhesion	AKT1, EGFR, IGF1R, GSK3B, MET, RHOA, IGF1, SRC, KDR	2.77E-05
hsa05205	Proteoglycans in cancer	AKT1, EGFR, IGF1R, FGFR1, ERBB4, MAPK14, MET, RHOA, ESR1, MDM2, IGF1, SRC, KDR	1.35E-09
hsa05200	Pathways in cancer	AKT1, EGFR, FGFR2, FGFR1, AR, BMP2, RXRA, MET, PPARG, IGF1, STAT1, CDK2, IGF1R, GSK3B, RHOA, MDM2, GSTP1	4.03E-10
hsa05215	Prostate cancer	AKT1, FGFR2, EGFR, IGF1R, FGFR1, AR, GSK3B, MDM2, IGF1, CDK2	1.91E-09
hsa04066	HIF-1 signaling pathway	AKT1, EGFR, IGF1R, IGF1, HK1, NOS3	2.70E-04

## Data Availability

The data used to support the findings of this study are included within the article and the supplementary information files.

## Supplementary Materials

Supplementary Materials Supplementary 1. Supplementary Table 1: the information of active compounds in DSLHG formula.* Supplementary 2.* Supplementary Table 2: the 192 potential targets of active ingredients in DSLHG formula.* Supplementary 3. *Supplementary Figure 1: HE and Masson staining of liver sections for each group.
