# Supplementary figures and images for: Network Pharmacology-Based Prediction of the Active Compounds, Potential Targets, and Signaling Pathways Involved in Danshiliuhao Granule for Treatment of Liver Fibrosis
Source: Evid Based Complement Alternat Med. 2019 Jul 3;2019:2630357. doi: 10.1155/2019/2630357 (PMC6636523; doi:10.1155/2019/2630357)

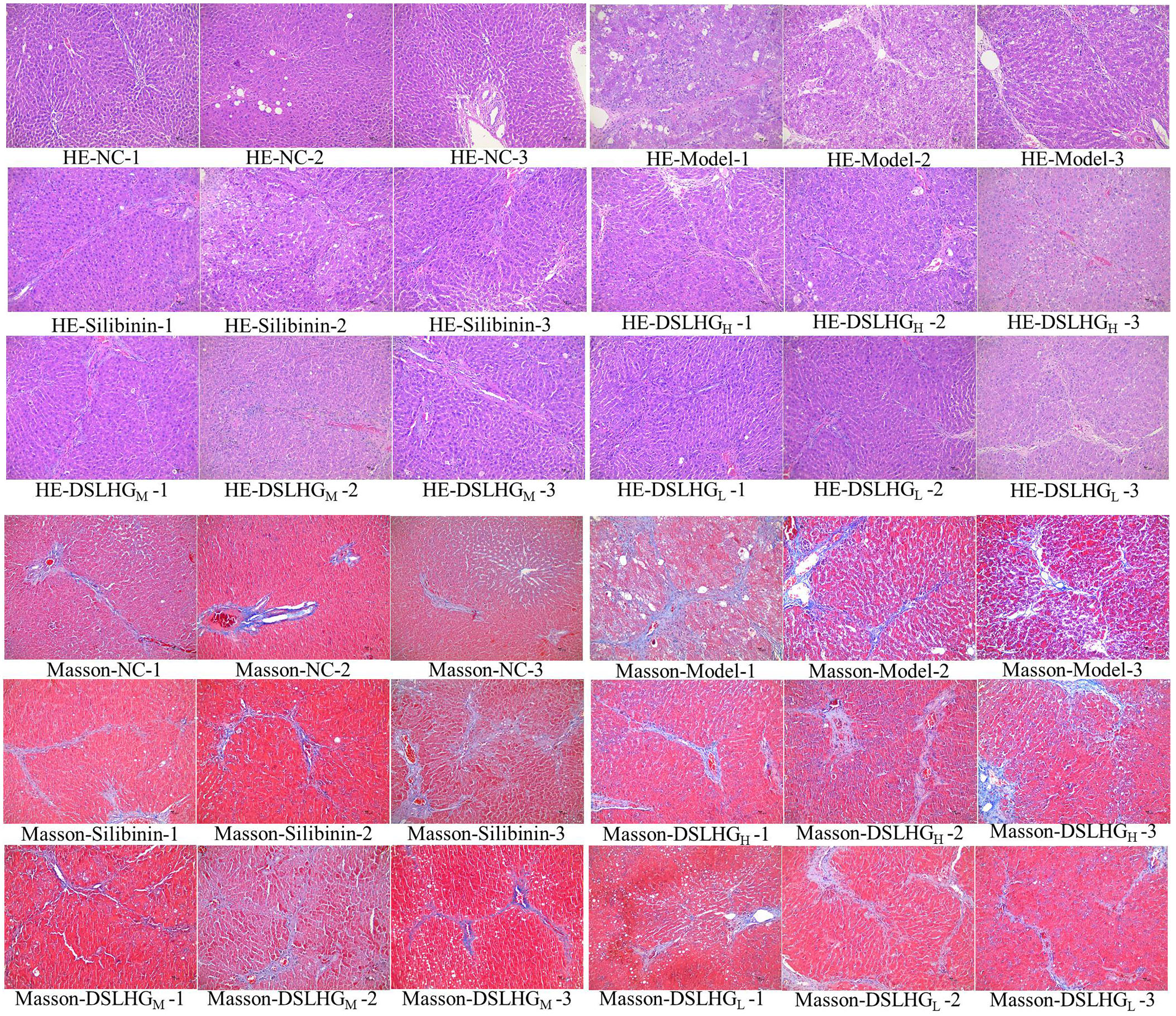

Supplement: Supplementary Materials — Supplementary 1. Supplementary Table 1: the information of active compounds in DSLHG formula. Supplementary 2. Supplementary Table 2: the 192 potential targets of active ingredients in DSLHG formula. Supplementary 3. Supplementary Figure 1: HE and Masson staining of liver sections for each group. [file 2630357.f1.zip › 2630357.f1/Supplementary Figure 1.jpg]
